# GPR-TSBiNet: An Information Gradient Enrichment Model for GPR B-Scan Small Target Detection

**DOI:** 10.3390/s25072223

**Published:** 2025-04-01

**Authors:** Chongqin Wang, Yi Guan, Minghe Chi, Feng Shen, Zhilong Yu, Qingguo Chen, Chao Chen

**Affiliations:** 1 School of Electrical and Electronic Engineering, Harbin University of Science and Technology, Harbin 150080, China; 18045629231@163.com (C.W.); chiminghe1985@hrbust.edu.cn (M.C.); zlyu@hrbust.edu.cn (Z.Y.); qgchen@263.net (Q.C.); 2 School of Instrumentation Science and Engineering, Harbin Institute of Technology, Harbin 150001, China; fshen@hit.edu.cn; 3 College of Materials Science and Engineering, Taiyuan University of Technology, Taiyuan 030024, China; chenchao@tyut.edu.cn

**Keywords:** feature extraction, transformers, GPR, deep learning, optical imaging

## Abstract

Accurate detection of underground grounding lines remains a significant technical challenge due to their deep burial and small cross-sectional dimensions, which cause signal scattering in heterogeneous soil media. This results in blurred features in GPR B-scan images, impeding reliable target identification. To address this limitation, we propose GPR-TSBiNet, an architecture incorporating two key model innovations. We introduce GPR-Transformer (GPR-Trans), a multi-branch backbone network specifically designed for GPR B-scan processing. In the neck stage, we develop the Spatial-Depth Converted Bidirectional Feature Pyramid Network (SC-BiFPN), which integrates SPD-ADown to mitigate feature loss caused by traditional pooling-based downsampling. We employ Shape-IoU as the loss function to enhance boundary detail preservation for small targets. Comparative experiments demonstrate that GPR-TSBiNet outperforms state-of-the-art (SOTA) models YOLOv11 and YOLOv10 in detection accuracy, achieving an AP0.5 improvement of 11.6% over YOLOv11X and 27.4% over YOLOv10X. Notably, the model improves small-target APsmall to 49.4 ± 0.7%, representing a 13.4% increase over the SOTA YOLOv11 model. Finally, real-world GPR validation experiments are conducted, confirming that GPR-TSBiNet provides a reliable solution for underground grounding line detection in GPR-based target recognition.

## 1. Introduction

The underground grounding system of a substation is critical to the safe and reliable operation of the power grid. Certain underground facilities, especially grounding wires, may be broken due to seismic activities or external forces, seriously affecting the operation of the power grid. Therefore, regular inspection of underground grounding wires is essential to ensure stable power supply to the grid. The underground environment limits the methods of inspecting grounding wires, and problems such as improper installation can make target detection during maintenance difficult. As a result, traditional inspections usually require a lot of time and manpower, with the risk of missed inspections and even prolonged power outages. Ground Penetrating Radar (GPR), a non-destructive testing technique based on electromagnetic wave propagation, offers a new solution for regular inspection and maintenance of underground equipment. GPR has proven to be particularly effective in detecting targets in underground environments and can significantly improve earth wire detection. However, there is an inherent trade-off between depth of penetration and spatial and temporal resolution, and the detection of elongated metallic targets exhibiting weak scattering characteristics under high-clutter underground conditions using ground-penetrating radar is extremely difficult, with a large number of small, non-standard targets contained in the B-scan images produced for underground earth wire detection. The traditional GPR B-scan small target recognition ignores the problems of target irregularity, cluttered signals, blurred targets, and even small target loss brought about by depth deepening.

**Figure 1:**
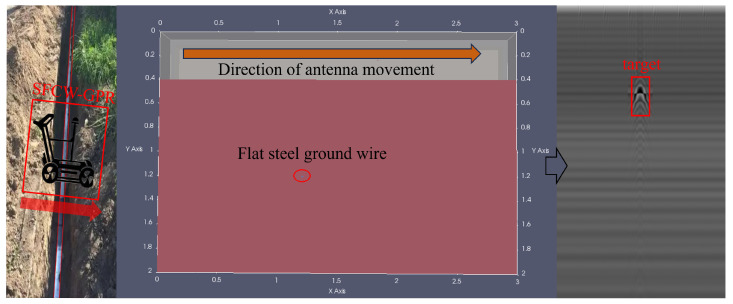
Schematic diagram of ground-penetrating radar detection and echo of underground grounding wire.

The above problems are particularly prominent in the detection of grounding lines, which makes targets in grounding line detection heavily dependent on the researcher’s expertise and interpretation skills, and may result in ignoring small target signals from grounding lines. There is an urgent need for more accurate and objective target identification methods to address the problem of neglected grounding system return signals and inconsistent identification results from different researchers. To restore the difficulty of grounding line detection by ground-penetrating radar, we simulate small target samples (smaller than 32 × 32 pixels in the B-scan image [1]) observed by ground-penetrating radar (GPR) during transverse scanning of the grounding system using gprMax to simulate the echo signals from the cross-section of the grounding line, as shown in Figure 1.

Numerous researchers have made significant contributions to the field of GPR B-scan image detection [2,3,4,5,6,7,8,9]. In terms of the application of GPR images in power systems, F.J. Prego et al. [10] used GPR to detect underground pipelines and created a dataset of the detection results; Zhou et al. [11] also performed underground cable detection and mapped the underground grounding system. Despite the success of these studies in detecting and locating underground grounding systems, the traditional detection models do not meet the needs when faced with the difficulties posed by the combined effects of depth and detection of target strips. In addition, some researchers have observed that B-scan images of GPR usually contain a large number of small target samples due to small target echo signals or long acquisition times. Liu et al. [12] proposed a feature-enhanced multi-scale visual transform method for road defect classification of GPR images, whereby images at different scales are detected and weighted to be fused to improve the classification accuracy. Multiscale feature fusion can improve the detection efficiency of small targets to a certain extent. Wang et al. [13] proposed a generalised underground pipeline recognition network ConvNeXt-YOLOv5 for GPR images, which significantly improved the recognition accuracy of small targets in B-scan images. However, the method’s utilization of multi-scale feature fusion and dilation convolution to enhance feature extraction for small target samples is not accompanied by a specific analysis of the detection of small targets. Additionally, it lacks a method to address the problems of information bottleneck and the loss of feature information caused by the downsampling process.

To address the issue of numerous small-to-medium target samples in GPR B-scan detection of grounding systems, we explore methods from computer vision for small target detection. We categorize these optimization methods into three types based on model enhancement approaches.

The first category of optimization methods involves the use of new Intersection over Union (IoU) metrics to enrich bounding box information [8,14,15,16] and F.J. Prego et al. [10]. This approach improves small target detection by incrementally increasing the IoU threshold to obtain high-quality positive samples [17,18]. Li et al. introduced CIoU [19], which combines normalized Gaussian–Wasserstein distance with a regression loss function; Huang et al. proposed IA-CIoU [20], which effectively controls auxiliary bounding box generation through a scaling factor. These improvements significantly enrich the information within predicted bounding boxes, thereby enhancing detection accuracy. Although the aforementioned models have made progress in detection results by optimizing IoU, excessively high IoU thresholds may reduce the number of matching anchors, leading to missed detections. Therefore, increasing the IoU threshold to improve small target detection accuracy is not advisable.

The second category of optimization methods focuses on enriching the feature information acquired by the model, including enhancing input feature information, enriching information gradients, and reducing feature information loss [21,22,23,24]. Enriching the feature information obtained by the detection head allows for more accurate image processing. Li et al. [25] proposed a Contextual Feature Integration Module (CFIM) to extract implicit clues co-occurring with the object, compensating for the lack of features in small, weak objects. Guo et al. [26] proposed a new infrared ship target detection algorithm, YOLO-IRS, which incorporates the Swin Transformer to enhance feature extraction from infrared ship images, improving detection accuracy while maintaining measurement speed. Zheng et al. [27] proposed ESL-YOLO for remote sensing small targets, integrating feature enhancement, fusion, and a local attention pyramid module to enrich the feature information acquired by the model. The aforementioned methods enrich the feature information acquired by the model during small target detection, thereby providing higher resolution and a smaller receptive field for targeting small objects. Enriching the feature information acquired by the model undeniably enhances its detection accuracy for small targets; however, the increase in both information and computational processes inevitably leads to an increase in computational load. Therefore, it is not advisable to simply add more input information to the model.

The third category of optimization methods focuses on reducing the loss of feature information during transmission and computation. To reduce the computational load, most models employ pooling and other downsampling techniques. However, pooling inevitably leads to the loss of targets with only a few pixels, and some researchers have studied methods to address information loss during the model’s operation. Zhang et al. [28] proposed GLPool, a globally learnable pooling operation designed to enhance distinctive high-level features in global regions. Rachid Riad et al. [29], addressing the challenges of low resolution and small target visual tasks, proposed replacing stride convolution and pooling layers. Building upon this, Qiang et al. [30] proposed the SPID module to reduce the loss of spatial local information by bringing spatially adjacent pixels closer in the channel dimension. In addition to information loss during downsampling, the loss of feature information as the model deepens is also significant. Wang et al. introduced Programmable Gradient Information (PGI) [31] and integrated it into the auxiliary branch of YOLOv9, successfully addressing the information bottleneck issue. However, although the aforementioned methods propose various techniques to reduce feature information loss, they do not fully utilize the results obtained from each processing step. Fully integrating information from multiple scales can improve small target detection accuracy in GPR B-scan while avoiding a significant increase in computational load.

In response to the problems mentioned above, we have developed a novel information-rich model, GPR-TSBiNet. This model was designed with the objective of addressing the challenge of detecting highly doped, low-resolution, and low-recognition B-scan targets obtained by ground-penetrating radar in the presence of ground-line detection. Furthermore, this model aims to minimize model computation while maximizing the feature information extracted from small targets by our model.

**Figure 2:**
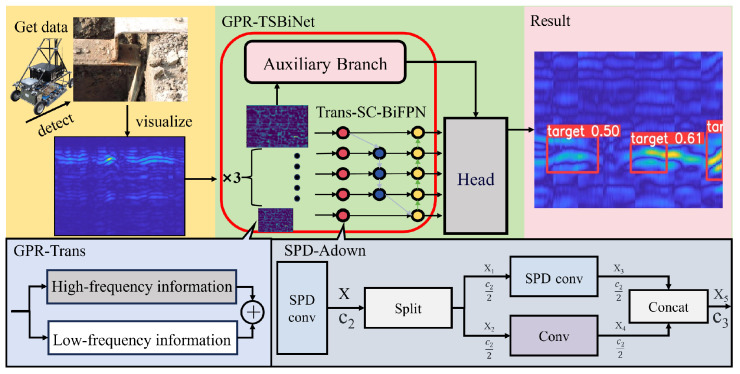
The main content of GPR-TSBiNet.

The key contributions of this paper are as follows:

1. A new network architecture GPR-TSBiNet has been designed specifically for B-scan detection of small underground targets, which can effectively improve the accuracy of ground-penetrating radar detection results. The structure of the model is shown in Figure 2.
2. Two modules, GPR-Trans and SC-BiFPN, are proposed to enhance the feature information of the model and mitigate feature information loss caused by the loss of Information and information bottlenecks.
3. An ultra-wideband stepped-frequency ground-penetrating radar (GPR) dataset, named Sub-GPR, designed specifically for substation underground grounding line detection, is constructed, with small target samples constituting 45.7% of the dataset.
4. Pre-training is conducted on the small target parabolic dataset and transfer learning is used to apply the learned weights to the Sub-GPR dataset, significantly improving the response time and accuracy of the model in detecting small target GPR samples.
5. We validate the detection results of the model in laboratory conditions, and the results demonstrate a significant improvement of our model in detecting grounding lines in power systems compared to other models.

In summary, our model achieves a 11.6% improvement in the AP0.5 metric on the GPR dataset for underground grounding lines in power systems compared to the state-of-the-art model YOLOv11, a 27.4% improvement over YOLOv10, and a 9.8% improvement over YOLOv9, demonstrating superior performance in detecting complex targets in GPR B-scans.

## 2. Methods

**Figure 3:**
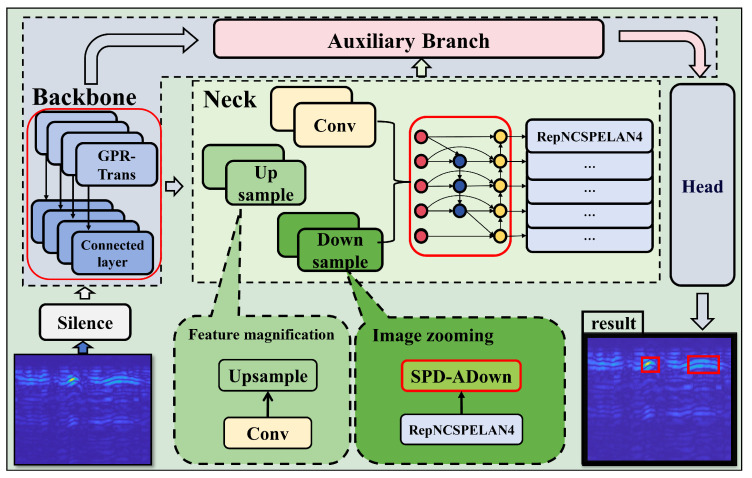
Schematic diagram of structure of GPR-TSBiNet.

**Figure 4:**
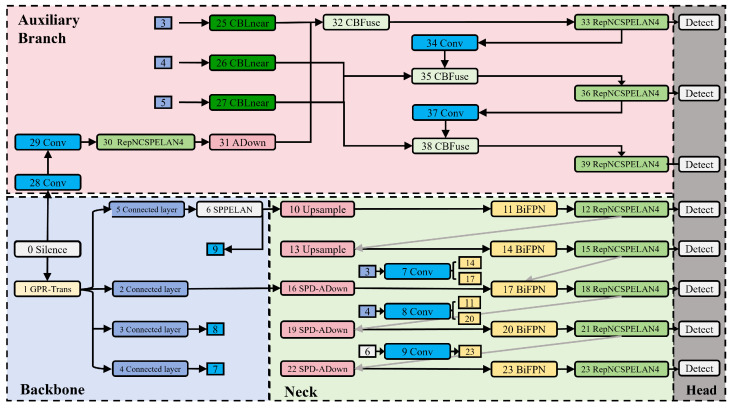
Overall framework of GPR-TSBiNet. The blue, green, pink, and gray areas represent the backbone, neck, auxiliary branch, and head of the model, respectively.

In this section, we comprehensively analyze the GPR-TSBiNet architecture, systematically introduce the GPR-Trans and SC-BiFPN modules, and discuss their underlying principles. To enhance detection accuracy across various pixel targets in GPR B-scan datasets and reduce the detection omissions of small targets, which account for a significant portion of the dataset, we propose the novel GPR-TSBiNet model to address the issue of information loss related to small targets in deep models. To address this issue, we introduce two strategies aimed at minimizing the information loss of small target features: This study proposes a three-branch architecture as the backbone of the model. This architecture has three advantages: it enhances the local feature extraction capability, captures cross-scale image features, and solves the information bottleneck caused by increasing model depth. Also, this architecture enhances the sensory field of the model. In the neck, we introduce SC-BiFPN, which employs a method called Enhanced SPD to mitigate small target information loss caused by pooling during downsampling. To enhance the reliability of small target detection, we perform weighted fusion of outputs from scale transformations, allowing small targets lost during specific scale transformations to be preserved. The structure of GPR-TSBiNet is shown in Figure 3 and Figure 4.

### 2.1. GPR-Trans Backbone

**Figure 5:**
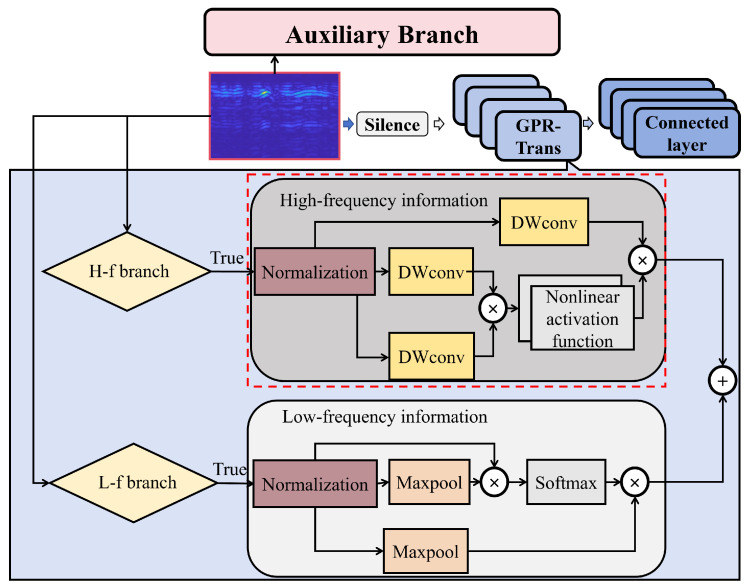
The three-branch GPR-Trans backbone consists of an orange-colored high-frequency feature extraction branch, a gray-colored low-frequency feature extraction branch, and a pink-colored auxiliary branch.

The backbone of the GPR-TSBiNet model is named GPR-Trans. Inspired by the VIT-Transformer [28] methodology, GPR-Trans employs a unique three-branch architecture. As the fundamental structural element, the backbone integrates multi-scale feature extraction and high- and low-frequency branches to enrich the model’s feature representation. It incorporates an auxiliary branch to address information bottlenecks, enabling effective feature extraction for small targets in GPR B-scans. The main components are illustrated in Figure 5.

(1) $$Q,K,V=\mathrm{FC}(X_{in})$$

where *Q*, *K*, *V* represent Query, Key and Value in the attention mechanism respectively, $X_{in}$ represents the input to the attention mechanism and **FC** represents the fully connected layer, we generate *Q*, *K*, *V* by linearly transforming the input.

(2) $$X_{lf}=Attn\left(Q_{l}f,\mathrm{Pool}\left(K_{l}f\right),\mathrm{Pool}\left(V_{l}f\right)\right)$$

where Attn represents the self-attention mechanism, $Q_{lf}$, $K_{lf}$, and $V_{lf}$ represent the low-frequency components of *Q*, *K*, and *V*, respectively, and **Pool** represents pooled downsampling. in the low-frequency branch, low-frequency global information improves the model’s target recognition accuracy. However, excessive low-frequency information not only overwhelms high-frequency effective features but also poses computational challenges. To address this, we process the low-frequency components using pooling, downsampling the low-frequency features of $K_{lf}$ and $V_{lf}$ to reduce the information volume of low-frequency data.

(3) $$V_{a}=\mathrm{DWconv}\left(V_{hf}\right)$$

where $V_{a}$ represents the output after aggregating high-frequency features, **DWconv** represents deep convolution, and $V_{hf}$ represents the high-frequency component of *V*. We perform deep convolution on $V_{hf}$ to aggregate high-frequency information, and it is worth noting that the weights of **DWconv** are shared globally.

(4) $$\begin{array}{cc} X_{hf} & =Attn⊙V_{a}\\ Q_{a} & =\mathrm{DWconv}(Q_{hf})\\ K_{a} & =\mathrm{DWconv}(K_{hf})\\ Attn_{t} & =\mathrm{FC}(\mathrm{Swish}\left(\mathrm{FC}\left(Q_{a}⊙K_{a}\right)\right))\\ Attn & =\mathrm{Tanh}(\frac{Attn_{t}}{\sqrt{d}}) \end{array}$$

where $Q_{a}$ and $K_{a}$ represent the high frequency feature information aggregation of *Q* and *K* respectively, $Q_{hf}$ and $K_{hf}$ represent the high frequency components of *Q* and *K*. Swish and Tanh are the nonlinear activation functions. To address the high-frequency components, it is necessary to introduce high-frequency branches and use **DWconv** to aggregate *Q* and *K* in these branches. The high-frequency components of $Q_{hf}$ and $K_{hf}$ are taken as a Hadamard product for obtaining the context-aware weights. This is followed by linear variations, which include fully connected and nonlinear activations. The process was then repeated to obtain higher-quality context-aware weights.

(5) $$\begin{array}{cc} \; & X_{C}=\mathrm{concat}\left(X_{lf},X_{hf}\right)\\ \; & X_{Out}=\mathrm{FC}\left(X_{C}\right) \end{array}$$

Finally, we connect the outputs of the two branches and obtain the final result after linear transformation. In the high-frequency information branch, depthwise separable convolution (**DWconv**) is utilized to reduce computational complexity while generating shared weights. Significantly, the intrinsic pooling operation is removed to reduce the computational load further. This approach is crucial for preserving small target information within the high-frequency branch. **DWconv** is employed because it can potentially retain the maximum amount of information from the original image. Additionally, **DWconv** is applied twice within the sub-model block to generate context-aware weights for multiscale feature fusion. Finally, a nonlinear activation function, which is specifically designed to enhance the nonlinearity of the attention mechanism, is integrated.

(6) $$I\left(X,X\right)\geq I\left(X,f_{\theta }\left(X\right)\right)\geq I\left(X,g_{\phi }\left(f_{\theta }\left(X\right)\right)\right)$$

where, *I* represents the mutual information, while *f* and *g* are the transformation functions, and θ and ϕ are their respective parameters. In the context of deep neural networks, $f_{\theta }$ and $g_{\phi }$ denote the operations of two successive layers, respectively.

The above equation indicates that as the number of network layers increases, the likelihood of original data loss also rises. However, in deep neural networks, parameters are adjusted based on network outputs and given targets by computing loss functions to generate new gradients. Nevertheless, deep gradients struggle to effectively backpropagate to shallow layers (i.e., gradient vanishing). PGI mitigates this issue by programmatically controlling gradient generation and flow paths, ensuring that optimization objectives at different feature levels align with task requirements. Consequently, models employing deep networks tend to lose more complete information about predictive targets, potentially leading to the use of incomplete data during training.

In GPR-based grounding line detection, the presence of numerous small targets and noise complicates recognition. To address the challenges in B-scan image detection, the backbone network integrates attention mechanisms and feature fusion layers. However, this inevitably increases model depth, leading to cumulative errors as depth increases and resulting in deeper-level feature information loss. The gradient information, which is programmatically controlled, is incorporated into the auxiliary branch. Through backpropagation, it updates the gradients and weight parameters. Moreover, using reversible connections, it enhances the network information flow. Subsequently, multi-scale features are fused multiple times. This process further refines the features that are lost or overfitted as a result of scale transformations. Specifically, to some extent, this auxiliary branch overcomes the limitations of deep supervision, which is typically restricted to extremely deep networks. Deep supervision architectures typically involve multiple prediction branches tailored for different tasks, such as utilizing distinct feature pyramids to detect objects of varying sizes. However, linking the model to deep supervision branches may cause shallow-layer features to overemphasize small object detection, leading to significant loss of critical feature pyramid information required for accurate deep-layer object prediction.

To address this issue, the module inputs the output of each scale transformation into the feature fusion layer, ensuring that each feature pyramid receives comprehensive information about all target objects, thereby preserving the complete information required for performing various tasks.

### 2.2. SC-BiFPN Neck

**Figure 6:**
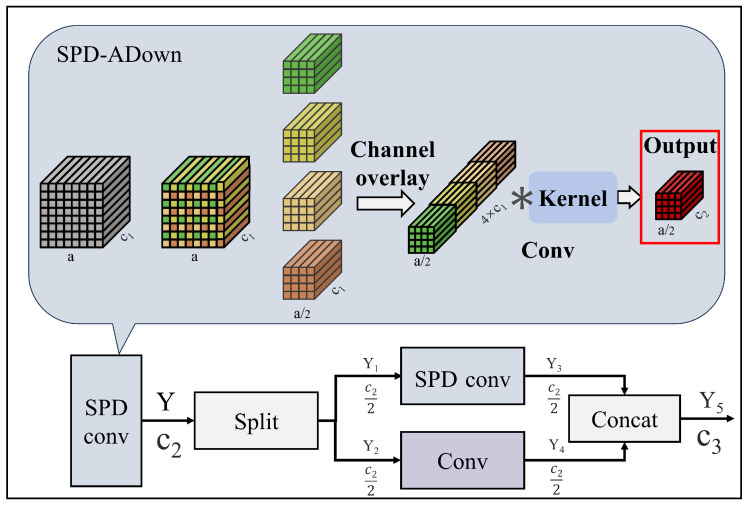
Schematic diagram of SPD-ADown, which uses channel convolution to replace pooling for downsampling.

In the neck component of GPR-TSBiNet, to more effectively integrate outputs from the GPR-Trans layer in the backbone, we introduce the SC-BiFPN structure to process and merge outputs of varying scales. The processed features of these varying scales are distributed across five distinct layers. The BiFPN structure establishes both top–down and bottom–up pathways. The top–down pathway facilitates feature fusion, enriching the predictive feature map with higher-level semantic information, which is expected to improve prediction accuracy. The bottom–up pathway transfers spatial information to the predictive feature map, ensuring it contains both rich semantic and precise location information. Finally, to minimize feature loss during the model’s scale transformation, we propose optimizing the downsampling process in BiFPN using a space-to-depth convolution approach. The Bidirectional Feature Pyramid Network (BiFPN) employs a weighted feature fusion method called Fast Normalized Fusion.

(7) $$O=\sum_{i}\frac{w_{i}\times I_{i}}{\epsilon +\sum_{j}w_{j}}$$

where $w_{i}$ represents the learnable weight, ϵ is a small weight for avoiding numerical instability, which is set to 0.0001. The weight in question has been meticulously calibrated to ensure that each standard weight is confined to intervals between 0 and 1. This weight functions as a quantitative metric, quantifying the relative importance of each input. In order to guarantee that wi is positive, a RELU activation function is incorporated following each $w_{i}$. This feature fusion approach has been demonstrated to balance accuracy and speed while exhibiting relatively modest GPU requirements during training. During the downsampling phase of SC-BiFPN, to preserve more original feature information and prevent the loss of critical small target features through pooling, we integrate the lightweight and flexible ADown module with SPD-conv. This approach aims to minimize small target information loss, with only a slight increase in computational overhead. The integration of SPD-Adown aligns with our objective of mitigating information loss in this study. To ensure that the aforementioned rich feature information is retained during downsampling, we replace the pooling layer in the original ADown with SPD. The combined downsampling structure and the underlying principles of the SPD layer are illustrated in Figure 6.

(8) $$\begin{array}{cc} f_{0,0} & =X[0:a:N_{s},0:S:N_{s}],\\ \cdots \\ f_{M_{s},0} & =X[N_{s}-1:a:N_{s},0:a:N_{s}];\\ f_{0,1} & =X[0:a:N_{s},1:a:N_{s}],\\ \cdots \\ f_{M_{s},1} & =X[M_{s}:a:N_{s},1:a:N_{s}];\\ f_{0,M_{s}} & =X[0:a:N_{s},M_{s}:a:N_{s}],\\ \cdots \\ f_{M_{s},M_{s}} & =X[M_{s}:a:N_{s},M_{s}:a:N_{s}]. \end{array}$$

where *Y* represent the output of every layer. $c_{1}$ and $c_{2}$ represent the number of channels in the original feature map and the number of channels after SPD-conv transformation, respectively. $f_{x,y}$ represents the sub-feature maps, a represents the number of pixel points, $N_{s}$ represents the number of scales. The original feature map $X_{i,j}$ is divided into sub-feature maps $f_{x,y}$ by divisible *i* + *x* and *i* + *j* in a certain ratio. Subsequently, we stack these sub-feature maps along the channel direction to obtain a new feature map, X’, which has its planar dimension reduced by a factor of scale and its channel size increased to $scale^{2}$.

At the end of the SPD convolutional layer, we add a single-step-length convolution that transforms the channel size of X’ from $scale^{2}$$c_{1}$ to $c_{2}$. The purpose of this is to retain as much as possible all of the feature information that is useful for the detection after the channel is convolved, and to reduce the loss of feature information by downsampling while retaining downsampling to speed up the detection. The essence of the SPD-conv lies in dividing the image into integrable scales. It downsamples the image by superimposing the sub-scales across the channels, thereby reducing the image size and enriching the information channels. Finally, the channels are compressed using a single-step convolution to optimize the issue of increased computational load. The essence of the SPD-conv lies in dividing the image into integrable scales. It downsamples the image by superimposing the sub-scales across the channels, thereby reducing the image size and enriching the information channels. Finally, the channels are compressed using a single-step convolution to optimize the issue of increased computational load.

### 2.3. ShapeIOU

IoU plays a crucial role in accurately detecting small target samples in the training set. The traditional IoU method considers the influence of distance, shape, and angle between the GT frame and the Anchor frame on bounding box regression but overlooks the impact of the bounding box’s own shape and size. To minimize the effect of the bounding box on small targets, we employ ShapeIOU [32]. This bounding box regression loss function considers the effect of the shape and size of the bounding box regression sample on the bounding box regression. The Intersection over Union (IoU) is employed to augment the bounding box information. This augmentation enriches the positional information associated with the bounding and ground truth boxes. This approach helps reduce the impact of the bounding box on detection accuracy, thereby improving the detection accuracy of small target samples.

## 3. Dataset Preparation

In this section, we provide a detailed introduction to the Sub-GPR dataset, focusing on the equipment used, the dataset construction method, and an analysis of its characteristics.

### 3.1. Experimental Equipment

**Figure 7:**
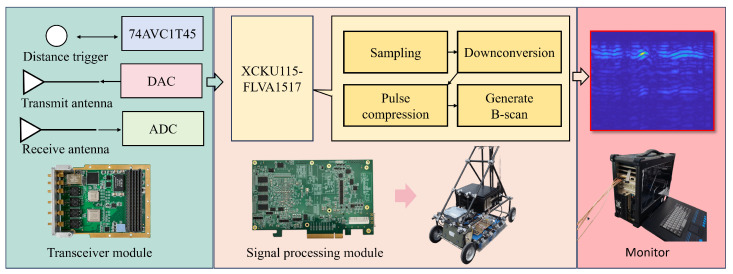
Schematic diagram of stepped-frequency ultra-wideband ground penetrating radar.

The dataset was generated using ultra-wideband stepped-frequency continuous-wave ground-penetrating radar, as depicted in Figure 7.

We utilized ADC + DAC FMC with high-sampling-rate sub-boards as the transceiver module and a PCIe 3.0 x8 carrier board as the signal processing module. The transmitter and radar receiver were constructed using direct digital frequency synthesis technology, and a baseband signal processing system was designed based on PC and FPGA. The Vivaldi antenna ensures that the digital signals emitted by different frequency bands maintain stable antenna gain as the signal changes. Through the signal link, clutter is suppressed and the transmit signal is amplified, enabling the equipment to operate within the 300 MHz to 1 GHz frequency band.

### 3.2. Data Acquisition

**Figure 8:**
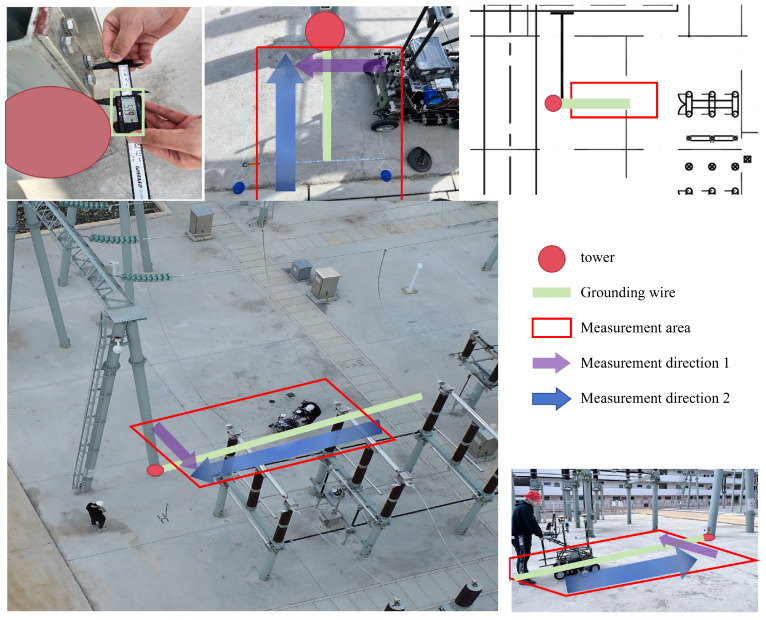
Data acquisition process. The red circle indicates the tower, the green line illustrates the schematic layout of the underground facilities, the red box marks the collection area, and the purple and blue arrows denote the vertical and horizontal collection directions, respectively.

The grounding line, being an underground facility with a small cross-section and extended erection distance, generates numerous small targets during the detection process using ground-penetrating radar. We employed ultra-wideband stepped-frequency continuous-wave ground-penetrating radar in a substation to detect underground facilities, as shown in Figure 8.

First, based on the underground facilities network map, we selected an area with sparse lines to test the reliability of radar detection for underground facilities. We then measured the grounding line in question. Finally, we conducted 8 experiments within a 2-m range around the grounding line, utilizing field grid block measurements to assess both the grounding line and the parallel grounding line as measurement directions. Additionally, to expand the dataset with more cases, we also conducted detections on the complex parts of the underground facilities throughout the substation.

### 3.3. Dataset Analysis

**Figure 9:**
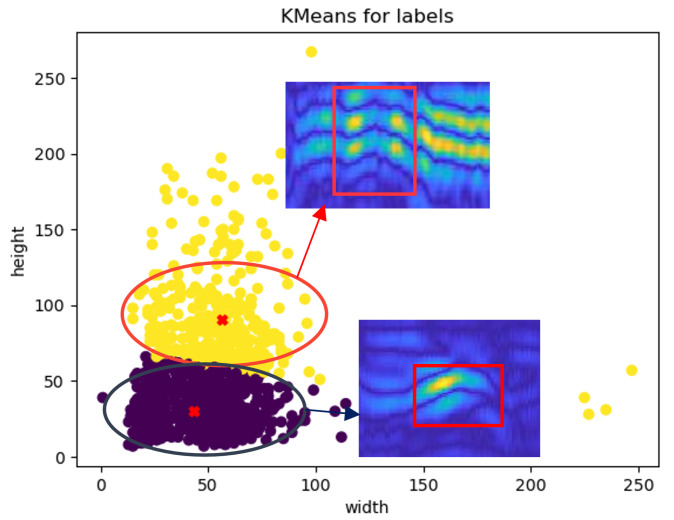
Distribution of object sizes in Sub-GPR, The dark part has a weak second echo and is not marked. The second echo in the yellow part is considered to be part of the feature.

There are 244 B-scans in Sub-GPR. Experiment 1, the Field Grid Validation Experiment, contributed 16 B-scans to the dataset, while the detection of the complex parts added 238 images. After labeling, the dataset contained a total of 1208 labels. According to the coco dataset definition, a small object is characterized by an image size less than 32 × 32 pixels. Our dataset includes 552 small objects. For analysis purposes, we clustered all the labels in the dataset after point tracing, as shown in Figure 9.

It was observed that most of the labels are concentrated in the 20×20−35×35 and 20×40−35×60 ranges. The detection results for the 20×40−35×60 range do not align with the actual status of the targets at our experimental site. Initially, the observed phenomenon engendered ambiguity. A rigorous investigation revealed that certain targets exhibit pronounced secondary backscatter signals within the ground-penetrating radar (GPR) dataset. These backscatter signals were erroneously assimilated into the target labels during the manual annotation process, as illustrated in the figure. This misclassification can lead to overestimating the target’s extent in the labeled data, which may have significant implications for subsequent data-driven analyses and model-based interpretations in GPR applications. However, we consider that the labeled secondary echoes significantly enhance the richness and size of the feature information. This dataset addresses the gap in GPR for detecting complex underground facilities in electric power systems. It highlights the challenges posed by the small cross-sections and the complex, diverse arrangements of underground facilities, thereby imposing stringent requirements on the small target detection capabilities of subsequent detection models.

## 4. Experiment

In this section, we assess the detection performance of GPR-TSBiNet on the GPR dataset through small target simulation tests and by comparing the results with other open-source models on the Sub-GPR dataset, highlighting the effectiveness of GPR-TSBiNet in identifying small targets in GPR data.

### 4.1. Training Setup

The training environment was built on an NVIDIA GeForce RTX 4090, with the software environment consisting of PyTorch v2.0.0 on Python 3.8 (Ubuntu 20.04) and CUDA 11.8. We set the model parameters to batch = 16 and epoch = 1000, with an early stopping patience of 50. This means that if the model parameters do not improve by more than 0.1% over fifty iterations, the training will be halted. It is noteworthy that in the experiments, due to the strong similarity between the hyperbola of the simulation and the actual ground-penetrating radar echo signals, we transferred the learning from the simulation experiments to the training of the ground-penetrating radar dataset. This approach aims to reduce training time and improve training accuracy.

### 4.2. Assessment Indicators

To evaluate whether the model meets the intelligent target recognition requirements of the ground-penetrating radar dataset, we evaluated the dataset using the following two detection speed metrics and five detection accuracy metrics. We evaluated the model using two detection speed metrics and five detection accuracy metrics. The detection speed metrics include parameters (Params) and FLOPs, while the detection accuracy metrics include precision (P), recall (R), average precision (AP), and average precision for small targets (AP(small)), which are evaluated on a separate small-target dataset to validate the effectiveness of our feature recognition. The equations for P, R, and AP are defined as follows:

(9) $$\begin{array}{cc} \mathrm{Precision} & =\frac{TP}{TP+FP}\\ \mathrm{Recall} & =\frac{TP}{TP+FN}\\ \mathrm{AP} & =\int_{0}^{1}P\left(R\right)dR \end{array}$$

where *TP* signifies the number of true positives, which are defined as positive samples that have been correctly identified as positive. *TN* denotes the number of true negatives, representing negative samples that have been accurately classified as negative. *FP* indicates the number of false positives, i.e., negative samples incorrectly classified as positive. *FN* denotes false negatives, which are negative samples incorrectly identified as positive. These four parameters are critical for assessing the accuracy of model identification.

### 4.3. Transfer Learning

**Figure 10:**
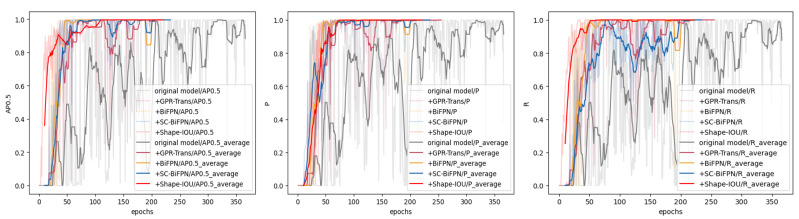
Ablation experiments on hyperbolic training of small targets.

To demonstrate the better performance of our model on datasets with a small target sample, we designed a smaller target sample to simulate the training of a real dataset, which has the advantage of being able to solve the complex phase noise, white noise, and echo interference in the dataset. We generated a dataset containing 100 hyperbolas with a pixel size ranging from 20 × 20 to 35 × 35, increasing in steps of 5. We split the GPR-TSBiNet model into different versions: the original model; one with GPR-Trans replacing the backbone; another with BiFPN added; and one with BiFPN downsampling replaced with SPD-ADown, named SC-BiFPN. Finally, a model with ShapeIoU added. We set the training parameters as follows: batch = 16, epoch = 1000, and patience = 50. These segmentation models were then used to perform resection experiments on the hyperbolic small object dataset, and the results are shown in Figure 10. We use an average sliding window to make the trend of the training results more obvious.

We selected AP0.5, precision (P), and recall (R) for comparison. It was determined that while all final models attained the highest AP0.5, the number of epochs necessary to achieve this AP0.5 differed. The original model reached its highest accuracy in 217 epochs, while the model with GPR-Trans achieved the highest accuracy in 183 epochs. The model with BiFPN reached the highest accuracy in 193 epochs, and the model utilizing SC-BiFPN achieved it in 152 epochs. Finally, our GPR-TSBiNet model achieved the highest accuracy in only 140 epochs during training. The figures further illustrate that our model enhances feature recognition and learning by reducing feature information loss, leading to better feedback during pre-training and requiring the fewest epochs to reach the highest accuracy.

### 4.4. Model Validation

**Figure 11:**
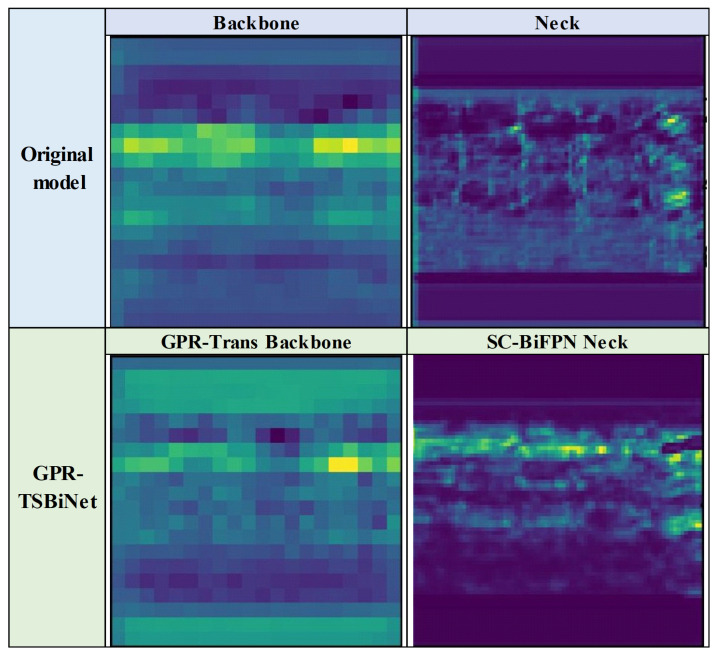
Influence of GPR-Trans and SC-BiFPN on feature extraction. The brighter color indicates that the model pays more attention to that area.

In order to accurately represent the contribution of our modules to the detection results, we performed ablation experiments and visualisation of module subfeature maps for each part in GPR-TSBiNet in the Sub-GPR dataset. First, to more intuitively reflect the contribution of GPR-Trans and SC-BiFPN to the prediction results, we adopt the feature map visualisation technique to visually reflect the changes in the feature maps after adding these two modules. We selected a B-scan with more obvious features to extract the feature maps of GPR-Trans and SC-BiFPN, respectively, during the training process, and YOLOv9’s Backbone and Neck feature maps are compared, as shown in Figure 11.

Compared to YOLOv9’s backbone and neck, GPR-TSBiNet’s feature map output aligns more closely with our labels in terms of both feature acquisition and feature shape. As the information is enriched, the model captures more comprehensive feature information, leading to more complete focus on relevant areas in the feature map. The target shape also becomes more prominent in the feature map.

**Figure 12:**
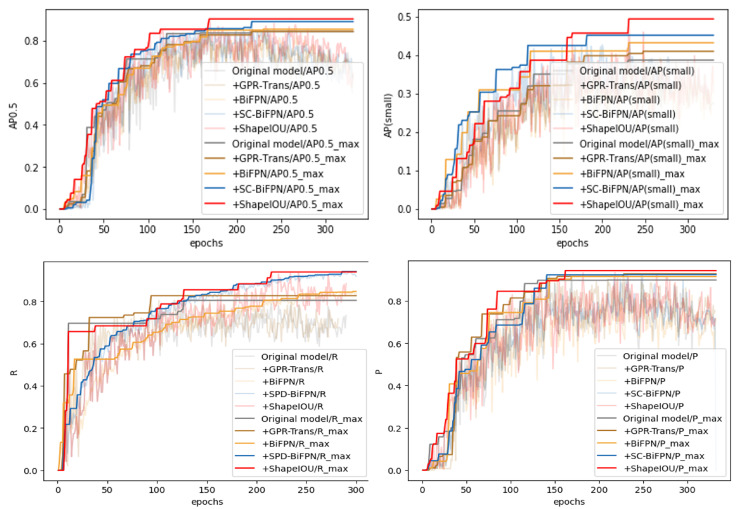
GPR-TSBiNet ablation experiments in Sub-GPR using the maximum-by-maximum method to visualise the best metrics obtained by the model.

Additionally, so as to further show that the inclusion of individual modules is effective for GPR-TSBiNet, we conducted ablation experiments on the model based on this dataset and the results are shown in Figure 12.

The incorporation of the GPR-Trans, SC-BiFPN, and ShapeIoU modules significantly enhances the AP, thereby illustrating the efficacy of the proposed model improvements. As shown in the figure, the improvements in AP, precision, AP(small), and recall clearly indicate an overall enhancement in model performance compared to the baseline model. Specifically, GPR-TSBiNet outperforms the original model, achieving improvements of 6.04%, 7.87%, and 1.27% in AP0.5, precision (P), and recall (R), respectively. However, while the aforementioned ablation experiments demonstrate the effectiveness of each module within the model when applied to the GPR dataset, the necessity of combining all modules has yet to be proven. To establish the importance of module integration, we designed additional ablation experiments that provide more direct insight into this necessity. We used Yolov9 as the original model, and sequentially integrated GPR-Trans, SC-BiFPN, and ShapeIoU in the order of backbone, neck, and head. The results of these additional ablation experiments, conducted on our constructed Sub-GPR dataset, are shown in Table 1.

The results of the ablation experiments on model combinations, including AP0.5, precision (P), and recall (R), are presented in Table 1. As shown in the table, the integration of the modules we constructed and selected progressively improves AP0.5, precision (P), and recall (R). Specifically, “O” denotes the original model, “G” refers to the GPR-Trans three-branch backbone, “S” stands for SC-BiFPN, and “I” represents ShapeIoU. Integrating each module improves the average accuracy, including GPR-Trans, SC-BiFPN, and ShapeloU, improving the model accuracy by 2.8%, 2.2%, and 1%, respectively. These results demonstrate that the combination of the modules outperforms their individual operation, thereby enhancing the detection of targets in GPR B-scans. For small target detection, our model achieves an AP0.5 (small) score of 0.49414% on the small target dataset, reflecting a 13.7% improvement over the baseline model, thereby validating the efficacy of our specialization in GPR small target detection. Small targets in GPR B-scans are typically only a few pixels in size, and insufficient feature information acquisition, along with information loss in the model, can lead to reduced detection accuracy for small targets. The extraction and fusion of multi-scale features enhance the model’s ability to represent small targets more effectively. Altering the downsampling procedure mitigates feature loss that results from pooling. The incorporation of auxiliary branches mitigates the information loss induced by the bottleneck effect resulting from the increased depth of the model. The enrichment of this information enhances the model’s detection accuracy. In terms of the computational complexity of the model, we can clearly see from the table that adding an attention mechanism to the backbone structure can reduce the use of the feature extraction fusion layer, thereby making our model lightweight in the backbone part. However, the parameters of the model have also increased because the IOU calculation of ShapeIOU is more computationally intensive than that of the original model. At the same time, the parameters of the feature pyramid using the SPD-ADown method have also decreased slightly compared to the original model. Overall, we have abandoned some feature extraction–fusion layers by using multi-scale feature fusion and a more accurate IOU function, with the aim of specifically extracting features for small objects and reducing some parameters.

### 4.5. Comparison

In order to compare the training results of GPR-TSBiNet in Sub-GPR, we selected the current SOTA models YOLOv11, YOLOv9, YOLOv10, and the past detection model YOLOv8. The results, based on the metrics Params, FLOPs, AP, AP (small), P (precision), R (recall), and F1 (F1-score), for these models are compared as shown in Table 2.

**Figure 13:**
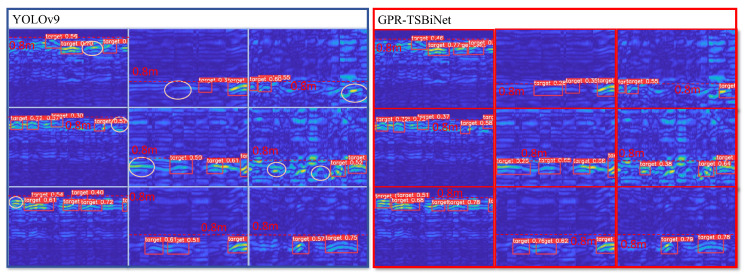
Comparison of detection results between YOLOv9 and GPR-TSBiNet models under the Sub-GPR dataset. The figure shows a dashed line at a depth of 0.8 m, and targets detected by GPR-TSBiNet but not YOLOv9 are circled.

The table clearly demonstrates that across the specified metrics, our model achieves superior detection accuracy for small underground facility targets in ground-penetrating radar (GPR), reaching 90.4%, which represents a 27.4% improvement over YOLOv10X and a 9.8% increase over YOLOv9E, and even 11.4% improvement over YOLOv11X. This validates that our approach to enriching feature information significantly improves the model’s detection accuracy. The average precision (AP (small)) of our model for small targets is also substantially improved compared to the other three models, further validating that the specialization for small targets in GPR-TSBiNet yields practical results in the training of Sub-GPR. Although we have employed several methods to enrich target information, leading to increased model computation, our model still outperforms YOLOv8X and YOLOv9E in terms of parameters and computational efficiency. This demonstrates that our use of SPD-conv in downsampling enhances the model’s accuracy without introducing a significant computational burden. Comparing P and R, it is evident that our model significantly reduces leakage and false detection rates when detecting Sub-GPR, indicating that our model offers a solution to the issue of high leakage and false detection rates in small target detection within underground facilities using GPR. We conducted a comparative analysis between the verification set produced by YOLOv9 training and that generated through GPR-TSBiNet training, as illustrated in Figure 13.

Targets identified by GPR-BiFPN but overlooked by YOLOv9 are highlighted using circular markers. It is evident that by enriching feature information, the model exhibits a significantly higher detection rate for B-scan images generated by GPR, especially when detecting underground narrow lines. When comparing the detection confidence levels between the two models, our model demonstrated an increase in confidence ranging from 1% to 5% over the original YOLOv9. This corroborates our hypothesis that minimizing the loss of feature information can effectively decrease the model’s missing rate and enhance detection accuracy.

## 5. Simulated Environment Verification Experiments

**Figure 14:**
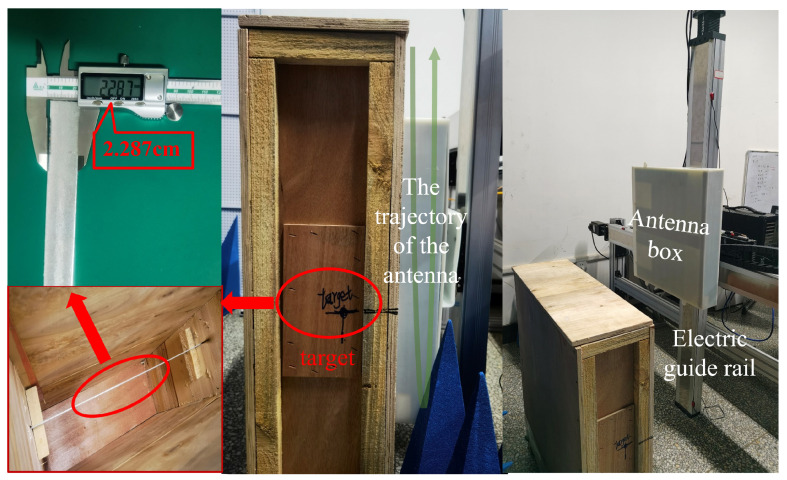
Grounding grid detection and verification experiment based on ground-penetrating radar.

To verify that our model can accurately detect small grounding line targets in B-scan images, we designed a validation experiment in which ground-penetrating radar (GPR) scans were carried out of underground grounding lines, followed by B-scan detection. First, we established a laboratory environment to simulate grounding line detection in substations, as illustrated in the Figure 14.

To replicate the underground environment of substation grounding lines, we constructed a sandbox using wooden boards and placed a 2.5 cm wide, 2 mm thick flat steel grounding line inside. The sandbox was secured with non-metallic materials and filled with fine non-metallic sand to ensure that the metal echo signals detected by the GPR originated solely from the grounding line. Additionally, we assumed a uniform dielectric constant for the sandbox filler. To control the GPR scanning path, we mounted the radar antenna on a guide rail, ensuring its movement along the Y-axis at the center of the sandbox. To enhance the credibility of the experiment, we performed two back-and-forth scans along the Y-axis from the top to the bottom of the sandbox, allowing the first and second experiments to serve as comparative references. The B-scan images obtained from both scans were input into the model for detection to evaluate its accuracy in grounding line identification. In both experiments, four targets of similar shape and size were detected in the B-scan images. Measurements confirmed that these targets had a resolution smaller than 35 × 35 pixels. The B-scan images from both experiments were fed into YOLOv11X, YOLOv10X, YOLOv9E, YOLOv8X, and our proposed model for target identification.

**Figure 15:**
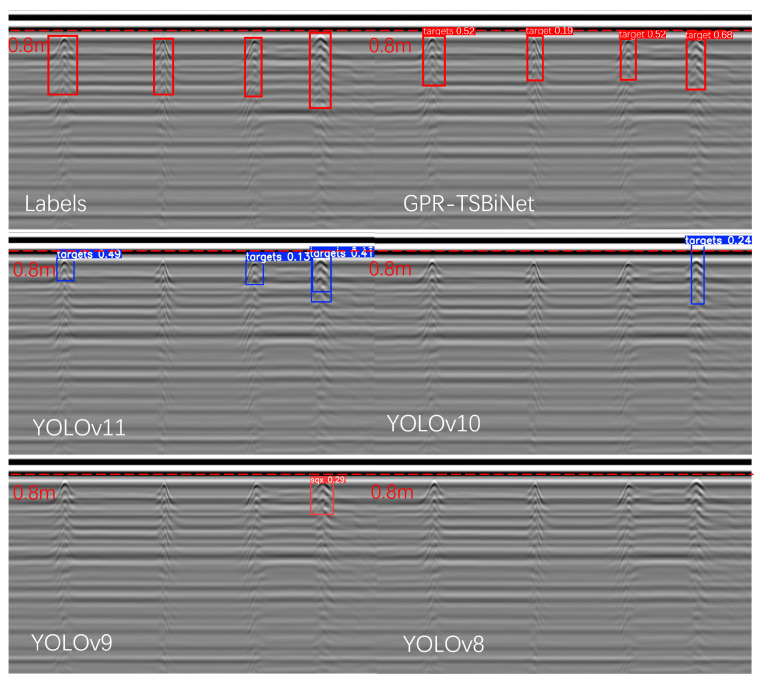
Verify the recognition results of B-scans collected in the experiment in different models, with the label as the control group.

As shown in Figure 15, among the four models tested on real-world targets, YOLOv8X achieved relatively high average precision when processing the dataset but performed poorly in detecting new B-scan images, failing to identify any targets. Both YOLOv9E and YOLOv10X identified the final target, though YOLOv9E exhibited a slightly higher detection confidence than YOLOv10X. YOLOv11X achieved the best performance in the B-scan images obtained from the laboratory validation experiment, successfully detecting the first, third, and fourth targets. It reached over 40% confidence in detecting the first and fourth targets. However, despite outperforming the other models, it still exhibited missed detections and low confidence scores for some targets. Our model successfully detected all targets, achieving confidence scores exceeding 50% for the first, third, and fourth targets. These results demonstrate that our model attains superior detection accuracy for small targets in GPR B-scan images, outperforming other models with similar parameter scales.

## 6. Conclusions

In this study, we designed a target recognition model for GPR B-scan imaging of underground grounding lines in power systems. We proposed an information-rich detection model, GPR-TSBiNet. It addresses the challenge of high false negative and false positive rates caused by the abundance of small target samples in GPR echo images. The model uses a three-branched GPR-Trans backbone. The low-frequency branch enhances contextual awareness and expands the model’s receptive field, while the high-frequency branch specializes in detecting targets embedded in high-frequency image features. In attempting to solve the information loss due to bottleneck effects, we developed an auxiliary branch that is designed to update information gradients and facilitate information flow. In order to integrate the multi-scale features of the GPR-Trans module and reduce small target feature loss due to downsampling, we proposed SPD-ADown, a downsampling approach incorporating SPD, and embedded it into the SC-BiFPN. Subsequently, to validate the reliability of our model, we employed ultra-wideband stepped-frequency GPR in substations to detect underground facilities and constructed a dataset. Finally, to assess the effectiveness of our detection model in real-world applications, we established a simulated underground grounding line environment in the laboratory. Comparative analysis demonstrated that our model achieves superior detection accuracy in GPR B-scan imaging of underground grounding lines in power systems. Extensive experiments confirm that the proposed GPR-TSBiNet model significantly enhances small target detection accuracy in GPR while reducing false negative and false positive rates.Compared to the state-of-the-art (SOTA) models YOLOv11 and YOLOv10, our model improves the AP0.5 metric by 11.3% and 27.4%, respectively.

## Figures and Tables

**Table 1:** Ablation experiment results.

Model	Param (M)	FLOPs (G)	AP0.5	P	R
Original model	69.3	243.3	0.844	0.897	0.758
GPR-Trans	52.8	224.2	0.852	0.798	0.812
SC-BiFPN	57.6	231.1	0.861	0.872	0.828
ShapeIoU	59.5	244.3	0.851	0.789	0.878
O+G	54.1	228.7	0.872	0.849	0.828
O+S	58.3	239.3	0.879	0.865	0.874
O+I	60.4	252.3	0.883	0.874	0.869
O+G+S	56.7	229.1	0.894	0.897	0.871
A+G+I	61.8	247.5	0.892	0.879	0.856
**GPR-TSBiNet**	**57.3**	**232.5**	**0.904**	**0.914**	**0.891**

**Table 2:** Comparison with other detection models in sub-gpr training.

Model	Params (M)	FLOPs (G)	AP0.5	AP (small)	P	R	F1
**GPR-TSBiNet**	**57.3**	**232.5**	**0.904**	**0.49414**	**0.914**	**0.891**	**0.896**
YOLOv8-X	68.2	257.8	0.897	0.451	0.893	0.872	0.882
YOLOv9-C	25.3	102.1	0.844	0.357	0.885	0.758	0.831
YOLOv11-L	25.3	86.9	0.842	0.346	0.897	0.758	0.822
YOLOv8-L	43.7	165.2	0.832	0.434	0.838	0.841	0.839
YOLOv9-M	32.6	130.7	0.829	0.384	0.881	0.761	0.817
YOLOv11-N	2.6	6.5	0.816	0.349	0.907	0.892	0.899
YOLOv9-E	69.3	243.3	0.806	0.383	0.830	0.717	0.769
YOLOv11-X	56.9	194.9	0.788	0.360	0.856	0.826	0.840
YOLOv11-M	20.1	68.0	0.751	0.305	0.834	0.804	0.819
YOLOv10-B	20.4	92.0	0.687	0.238	0.660	0.696	0.677
YOLOv10-L	25.7	120.3	0.682	0.310	0.776	0.565	0.654
YOLOv10-X	31.6	160.4	0.630	0.277	0.571	0.637	0.602

## Data Availability

Dataset available on request from the authors.
